# Treatment target re-classification of subjects comparing estimation of low-density lipoprotein cholesterol by the Friedewald equation and direct measurement of LDL-cholesterol

**DOI:** 10.1080/03009734.2018.1465496

**Published:** 2018-05-10

**Authors:** Anders Larsson, Emil Hagström, Lennart Nilsson, Maria K. Svensson

**Affiliations:** a Department of Medical Sciences, Uppsala University, Uppsala, Sweden; b Uppsala Clinical Research Centre, Uppsala, Sweden; c Department of Medical and Health Sciences, Linkoping University, Linkoping, Sweden

**Keywords:** Direct LDL-C measurement, Friedewald equation, LDL-cholesterol, primary prevention, re-classification, secondary prevention

## Abstract

**Aims:**

To compare low-density lipoprotein cholesterol (LDL-C) values calculated by the Friedewald equation with direct LDL-C in patient samples and assess the possible impact on re-classification of LDL-C target values for primary prevention or high cardiovascular disease (CVD) risk (<2.5 mmol/L) and secondary prevention or very high CVD risk (<1.8 mmol/L). LDL-C is an important CVD risk factor. Over the last decade, there has been a change in laboratory methodology from indirectly calculated LDL-C with the Friedewald equation to direct LDL-C measurements (dLDL-C).

**Methods:**

Reported results for plasma triglycerides, total cholesterol, high-density lipoprotein-cholesterol, and dLDL-C from 34,981 samples analyzed in year 2014 were extracted from the laboratory information system, Uppsala University Hospital, Uppsala, Sweden.

**Results:**

dLDL-C was approximately 10% lower than the corresponding LDL-C results calculated by the Friedewald equation in both men and women. In subjects with triglyceride concentrations above 4 mmol/L (*n* = 1250) the same discordant pattern was seen as for the entire study population. Altogether 5469 out of 18,051 men (30.3%) and 4604 out of 16,928 women (27.2%) were down-classified at least one CVD risk category. A very small number of subject was up-classified, in total 37 out of 18,051 men (0.2%) and 28 out of 16,928 women (0.2%).

**Conclusions:**

The two LDL-C methods had a high concordance, but the direct LDL-C measurement consistently gave approx. 10% lower values, and this caused one-third of subjects to be re-classified as having a lower cardiovascular disease risk in relation to recommended LDL-C target values and decision limits.

## Introduction

Low-density lipoprotein cholesterol (LDL-C) is an established cardiovascular risk factor often used as a surrogate marker for cardiovascular outcomes in randomized clinical trials (1). Current cardiovascular disease (CVD) prevention guidelines are based on an overall cardiovascular risk assessment including LDL-C values, and these guidelines are also a trigger for intensity of treatment with lipid-modifying therapy (LMT). Treatment guidelines recommend a LDL-C target value of <2.5 mmol/L in primary prevention in high-risk patient populations including patients with diabetes and chronic kidney disease, and <1.8 mmol/L in secondary prevention in patients with documented very high risk and established atherosclerotic CVD (2).

For more than 25 years laboratories have reported LDL-C calculated from total cholesterol, high-density lipoprotein cholesterol (HDL-C), and triglyceride concentrations using the Friedewald equation (3). However, during the last decade there have been several changes in methodologies for lipid profiling. Firstly, there was a shift towards direct HDL-C methods instead of precipitation methods, and, secondly, there has also been a change from calculated LDL-C to direct measurement of LDL-C (dLDL-C) (4). However, there is a paucity of knowledge on the concordance between direct and calculated LDL-C measurements.

Thus, the aim of this study was to compare LDL-C results calculated by the Friedewald equation with direct LDL-C (dLDL-C) measurements and assess the concordance in a large unselected population. In addition, we wanted to assess a potential cardiovascular risk re-classification based on the methodology used to measure LDL-C values.

## Materials and methods

### Study population and design

This study is based on a large unselected population that had triglycerides, total cholesterol, HDL-C, and direct LDL-C (dLDL-C) measured in the same blood sample tube. All tests were performed at Uppsala University Hospital Laboratory, Sweden, on fresh plasma samples during 2014. The subjects were assessed for various clinical reasons, and the blood samples drawn in both primary and secondary care and data were later extracted from the laboratory information system (FlexLab, Tieto, Stockholm, Sweden). Only test reports with valid quantitative results for all measurements and a complete personal identity number from subjects were included. A total of 34,981 samples were included in the study. The study was approved by the local ethics committee (EC) (dnr 01/367).

### Laboratory analyses

Plasma triglycerides (7D74-21), total cholesterol (7D62-21), HDL-C (3K33-21), and dLDL-C (1E31-20) were analyzed on an Architect ci8200 (Abbott Laboratories, Abbott Park, IL, USA) with reagents from the same manufacturer. The total coefficient of variation for the methods were 1.9% at 0.84 mmol/L and 1.1% at 2.24 mmol/L for triglycerides, 0.7% at 3.3 mmol/L and 0.5% at 5.7 mmol/L for total cholesterol, 2.4% at 0.80 mmol/L and 0.8% at 2.24 mmol/L for HDL-C, and 0.9% at 1.61 mmol/L and 1.1% at 2.80 mmol/L for dLDL-cholesterol. The laboratory participates in the Swedish external quality assurance programs for triglycerides, total cholesterol, HDL-cholesterol, and LDL-cholesterol provided by Equalis (Uppsala, Sweden). The assays are also traceable to Centers for Disease Controls and Prevention (CDC) reference methods.

LDL-C in mmol/L was calculated as LDL-C = total cholesterol − HDL-C − 0.456 × total triglyceride concentration (Friedewald equation) (3).

### Re-classification

In order to study the impact of re-classification according to CVD risk categories the Friedewald calculation of LDL-C was used as the standard baseline value. The proportion of subjects re-classified by using dLDL-C measurements at the cut offs for primary prevention and high CVD risk (1.8 mmol/L) and secondary prevention and very high CVD risk (2.5 mmol/L) was assessed.

### Statistical analysis

Descriptive data are presented as median and interquartile range (IQR) or as indicated.

Univariate regression analyses were utilized to demonstrate relationship and concordance between the Friedewald equation and dLDL-C measurement. Re-classification was calculated using Excel 2013 (Microsoft, Seattle, WA, USA).

## Results

### Study population

In total, there were 34,979 samples—18,051 from men and 16,928 samples from women. The median age was similar in men and women (65 years). Lipid values in all subjects and stratified by sex are shown in Table 1. Women in this study had numerically higher concentrations of the cholesterol fractions but lower triglycerides.

**Table 1.** Age and lipid values in all subjects (*n* = 34,979) and stratified by sex (men, *n* = 18,051; and women, *n* = 16,928).

	All subjects	Men	Women
*n*	34,979	18,051	16,928
Age (years)	65 (54–72)	65 (54–72)	65 (54–73)
Total cholesterol (mmol/L)	5.10 (4.30–5.90)	4.80 (4.10–5.70)	5.3 (4.60–6.20)
Triglycerides (mmol/L)	1.41 (1.02–2.02)	1.47 (1.05–2.13)	1.36 (0.99–1.90)
HDL-C (mmol/L)	1.30 (1.00–1.50)	1.20 (0.90–1.40)	1.40 (1.20–1.70)
dLDL-C (mmol/L)	3.10 (2.40–3.80)	2.90 (2.30–3.70)	3.20 (2.50–4.00)
LDL-C (mmol/L)^a^	3.39 (2.71–4.18)	3.27 (2.61–4.03)	3.52 (2.85–4.32)
LDL-C/HDL-C^a^	2.64 (2.02–3.42)	2.80 (2.15–3.64)	2.48 (1.91–3.20)
dLDL-C/HDL-C	2.40 (1.80–3.10)	2.50 (1.90–3.30)	2.30 (1.70–2.90)

Data are median (interquartile range; IQR).

^a^LDL-C calculated by the Friedewald equation.

dLDL-C: direct low-density lipoprotein cholesterol measurement; HDL-C: high-density lipoprotein cholesterol; LDL-C: low-density lipoprotein cholesterol.

### Comparison of LDL-C concentrations calculated by the Friedewald equation versus dLDL-C

**Figure 1.**
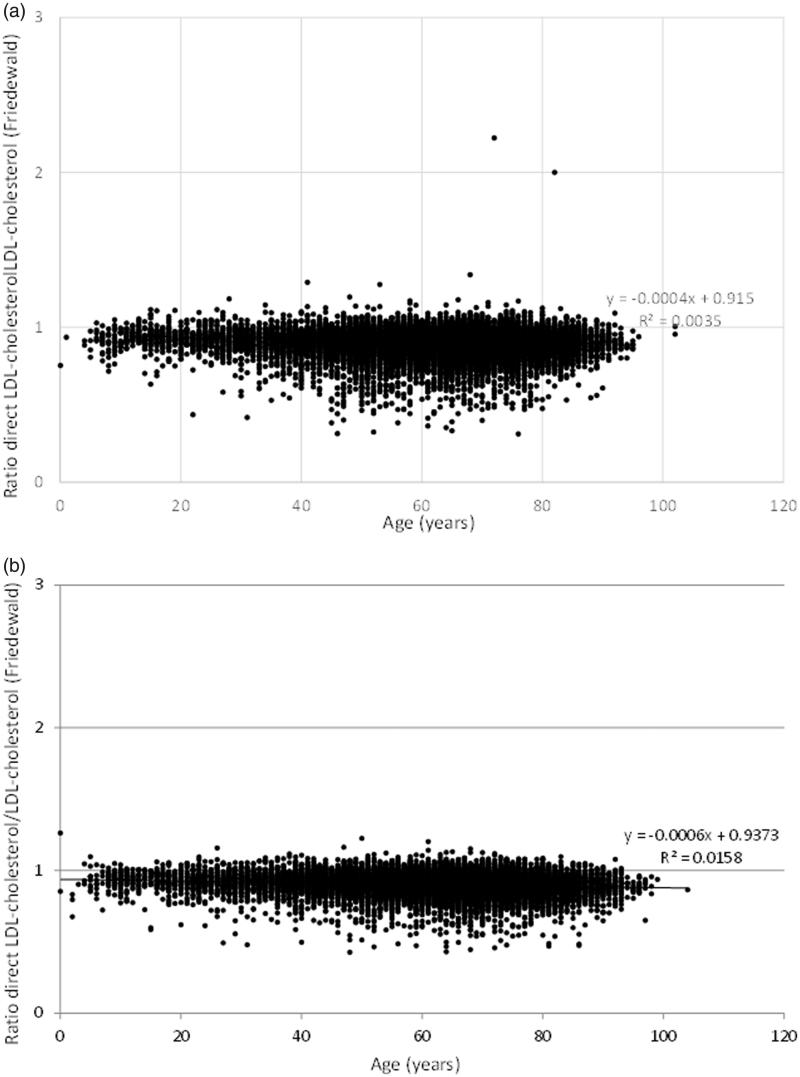
Age versus the ratio between direct LDL-C (dLDL-C) and LDL-C calculated with the Friedewald equation in (a) men (*n* = 18,051) and (b) women (*n* = 16,928). All results are presented.

**Figure 2.**
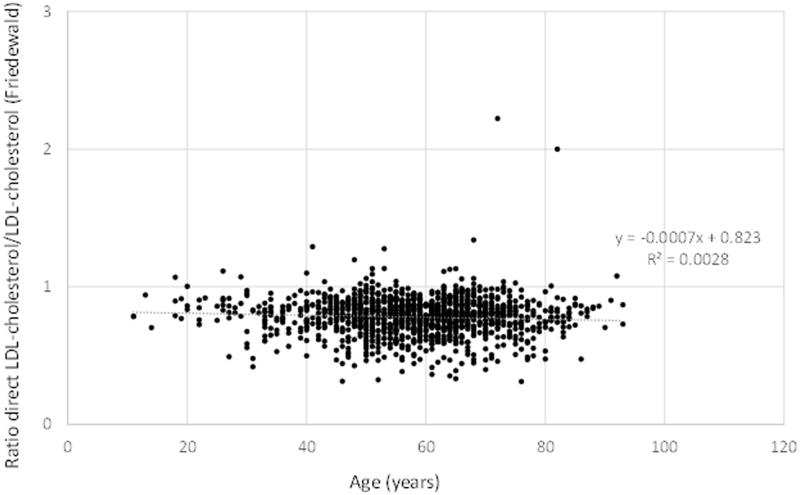
Age versus the ratio between direct LDL (dLDL) and LDL-C calculated with the Friedewald equation in subjects with triglycerides (TG) > 4.0 mmol/L (*n* = 1250). All results are presented.

Overall, the median dLDL-C measurement was approximately 10% lower (10.9% for males and 9.9% for females) than LDL-C calculated using the Friedewald equation in both men (Figure 1(a)), women (Figure 1(b)), and in all age groups (data not shown). In subjects with triglyceride concentrations >4 mmol/L (*n* = 1250) there was the same discordant pattern in relation to age as for the entire study population (Figure 2).

### CVD risk category re-classification dependent on the methodology used to measure LDL-C concentrations

**Figure 3.**
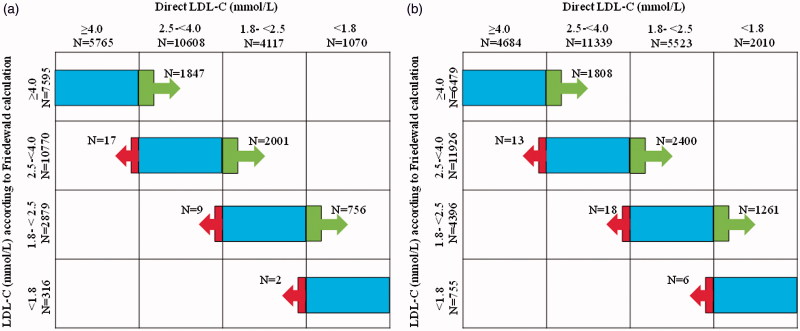
The number of (a) men (*n* = 18,051) and (b) women (*n* = 16,928) re-classified by direct LDL-C (dLDL-C) in comparison with LDL-C calculated with the Friedewald equation.

Up to one-third of subjects were down-classified in cardiovascular risk category in both men and women when using the dLDL-method as compared with the Friedewald equation (Table 2). In total, 5469 out of 18,051 men (30.3%) and 4604 out of 16,928 women (27.2%) were down-classified at least one category. A very small number of subject were up-classified, in total 37 out of 18,051 men (0.2%) and 28 out of 16,928 women (0.2%) (Figure 3).

**Table 2.** Number of men and women re-classified using direct low-density lipoprotein cholesterol measurements (dLDL) at the cut offs for primary prevention and high CVD risk (2.5 mmol/L), and secondary prevention and very high CVD risk (1.8 mmol/L), respectively.

Categories of LDL-C (mmol/L) calculated by the Friedewald equation	Number of subjects	Number of subjects down-classified using dLDL	Number of subjects up-classified with dLDL
Men (*n* = 18,051)			
≤1.8	749	0	6
1.8–2.5	3118	1261	18
2.5–4.0	9513	2400	13
>4.0	4671	1808	0
Women (*n* = 16,928)			
≤1.8	314	0	2
1.8–2.5	2114	756	9
2.5–4.0	8752	2001	17
>4.0	5748	1847	0

LDL-C: low-density lipoprotein cholesterol; dLDL-C: direct low-density lipoprotein cholesterol measurements.

## Discussion

In this study of unselected and consecutively analyzed lipid profiles, dLDL-C consistently showed approximately 10% lower values than LDL-C calculated using the Friedewald equation over the entire range of direct LDL-C studied (range 0.42–16.0 mmol/L). This discrepancy was also seen and was similar in subjects with triglycerides >4.0 mmol/L. Furthermore, if dLDL-C values were used for cardiovascular risk assessment and to aid LMT treatment intensity, almost one-third of the subjects were re-classified downward at least one treatment target value category. The proportion of subjects re-classified was similar in men and women.

Direct LDL (dLDL) measurement displayed approximately 10% lower values than values calculated using the Friedewald equation. This difference was similar in subjects with triglycerides >4.0 mmol/L and contradictory to the current tradition of reporting dLDL regardless of the triglyceride values while not reporting LDL-C values calculated by the Friedewald equation above a certain triglyceride value/threshold. In our opinion, this finding supports the recent statement by the European Atherosclerosis Society and the European Federation of Clinical Chemistry and Laboratory Medicine that fasting should not be required for LDL cholesterol testing (5). The difference found between the two LDL methods was independent of age and sex.

A 10% difference between the two methods could be interpreted as a relatively small difference, but this difference has a clear impact on the classification of subjects in relationship to the LDL-C target values for primary prevention or high CVD risk (<2.5 mmol/L) and secondary prevention or very high CVD risk (<1.8 mmol/L). In this study we are able to show that approximately one-third of all subjects (30% of the men and 27% of the women) were down-classified at least one category with dLDL when compared with Friedewald-calculated LDL-C. It is therefore our opinion that this difference is too large to be acceptable as the current treatment guidelines and recommendations are not laboratory method-specific when it comes to LDL-C measurements. The risk is that a substantial proportion of subjects may receive an inappropriately low intensity of LMT or in some cases no LMT at all.

A previous study by Martin et al. compared Friedewald-estimated LDL-C and LDL-C with density gradient ultracentrifugation for LDL-C determination (6). The authors reported that both the Friedewald equation and the directly measured LDL-C underestimated LDL-C, especially in patients with low LDL-C. This resulted in re-classification of a large number of patients from ≥70 mg/dL (≥1.8 mmol/L) to <70 mg/dL.

The Friedewald formula was introduced approximately 50 years ago (3). Even if the equation was accurate when it was originally introduced, several changes in laboratory methodology have occurred since the original publication. For instance, in the original publication by Friedewald et al., HDL-C, LDL-C, and VLDL-C were obtained by a combination of ultracentrifugation and precipitation procedures. Over the decades a number of different precipitation techniques have been used for HDL-C determination, and today all Swedish routine laboratories use direct HDL-C methods. These method changes are likely to introduce bias that will influence the performance of the original Friedewald equation in combination with these new methods. It thus seems reasonable to revalidate both the dLDL-C methods and Friedewald-estimated LDL-C. Martin et al. showed for instance that an adjustable factor for the TG:VLDL-C ratio provided more accurate guideline risk classification than the original Friedewald equation (7).

The Friedewald equation has been considered unreliable in the presence of chylomicronemia and hypertriglyceridemia, findings that are prevalent in patients with metabolic syndrome or type 2 diabetes, although this has been challenged (8). Many laboratories have therefore used a triglyceride (TG) limit for reporting LDL-C calculated by the Friedewald equation. With direct LDL-C methods, the laboratories report LDL-C also for samples with high TG values. There has thus been a shift in methodology in LDL-C measurements and reporting towards direct determination of LDL-C without considering TG results, and we expect this shift to continue. Recently the European Atherosclerosis Society and European Federation of Clinical Chemistry and Laboratory Medicine made a joint statement that fasting should not be routinely required for determination of lipid profiles (5).

The gold standard for determination of different lipid fractions is ultracentrifugation, but this is expensive and cumbersome in clinical laboratory settings and thus not feasible in a clinical setting (9,10). Therefore, other methods are used, but importantly one has to remember that treatment guidelines and recommendations are not laboratory method-specific.

There are of course both strengths and limitations in this study. An important strength is that the study included a large unselected adult population and that all lipid analyses and measurements were done in the same blood sample tube. A study limitation is that the results are extracted directly from the laboratory system without having access to clinical characteristics of the patients from whom the samples were collected or information on statin therapy and clinical outcomes. This also means that we do not know the proportion of subjects that actually had the different LDL-C concentrations as treatment targets and thus would have had a change in the intensity of LMT in a real-world clinical setting.

In conclusion, the method shift from LDL-C calculated by the Friedewald equation to direct LDL-C leads to the re-classification of one-third of subjects as having a lower cardiovascular disease risk in relation to recommended LDL-C target values and decision limits.

This re-classification will occur in both men and women and is independent of age. This problem needs to be addressed by adjusting the calibration of LDL-C methods to achieve a better agreement between Friedewald-calculated LDL-C and direct LDL-C. Whether this re-classification translates into a different clinical management and ultimately to different risk of atherosclerotic outcomes remains unknown and needs to be further evaluated.

## Disclosure statement

None of the authors has any conflict of interest. Maria K. Svensson is currently employed by Amgen AB.
